# Postmenopausal Obesity and Dyslipidemia as Risk Factors for Breast Cancer in Korean Women: Analysis of a National Health Screening Cohort

**DOI:** 10.3390/jcm14217816

**Published:** 2025-11-03

**Authors:** Mi Jung Kwon, Joo Hee Kim, Dae Myoung Yoo, Kyeong Min Han, Nan Young Kim, Hyo Geun Choi, Jung Ho Park

**Affiliations:** 1 Department Pathology, Hallym University Sacred Heart Hospital, Hallym University College of Medicine, Anyang 14068, Republic of Korea; 2 Division of Pulmonary, Allergy, and Critical Care Medicine, Department of Medicine, Hallym University Sacred Heart Hospital, Hallym University College of Medicine, Anyang 14068, Republic of Korea; 3 Hallym Data Science Laboratory, Hallym University College of Medicine, Anyang 14068, Republic of Korea; 4 Laboratory of Brain and Cognitive Sciences for Convergence Medicine, Hallym University Sacred Heart Hospital, Anyang 14068, Republic of Korea; 5 Hallym Institute of Translational Genomics and Bioinformatics, Hallym University Medical Center, Anyang 14068, Republic of Korea; 6 Suseo Seoul E.N.T. Clinic, 10, Bamgogae-ro 1-gil, Gangnam-gu, Seoul 06349, Republic of Korea; 7 Division of Breast and Endocrine Surgery, Department of Surgery, Hallym University Sacred Heart Hospital, Hallym University College of Medicine, Anyang 14068, Republic of Korea

**Keywords:** breast neoplasms, obesity, dyslipidemias

## Abstract

**Background/Objectives**: Over the past two decades, the incidence of breast cancer has been increasing in Korea, which is potentially attributable to longer life expectancies, Westernized lifestyles, and declining fertility rates. However, the contributions of modifiable metabolic and behavioral risk factors in Asian populations remain underexplored. This study aimed to assess the associations between health-related factors and incidence of breast cancer in a large Korean cohort. **Methods**: We analyzed data from the Korean National Health Insurance Service and included women who underwent health screening in 2009. Cases of breast cancer diagnosed between 2010 and 2021 were identified using medical claims and registration codes. The breast cancer cases were matched to controls in a 1:4 ratio based on age, income, and region of residence. Conditional logistic regression was used to calculate the adjusted odds ratios (aORs) and 95% confidence intervals (CIs) for key exposures. **Results**: In total, 52,869 breast cancer cases and 211,476 matched controls were included. The peak age group at diagnosis was 50–54 years. Dyslipidemia was associated with a 12% increase in the risk of breast cancer across all age groups (95% CI, 1.10–1.14). In women ≥ 50 years of age, a dose–response relationship was observed between body mass index (BMI) and breast cancer risk: aORs were 1.04 (95% CI, 1.01–1.08) for overweight, 1.14 (95% CI, 1.11–1.17) for obesity class I (BMI ≥ 25 to < 30 kg/m^2^), and 1.33 (95% CI, 1.26–1.41) for obesity class II (BMI ≥ 30 kg/m^2^). Conversely, being underweight was associated with a decreased risk (aOR, 0.81; 95% CI, 0.74–0.89). No consistent associations were observed with alcohol consumption, cigarette smoking, or the presence of diabetes mellitus. **Conclusions**: Postmenopausal obesity and dyslipidemia contribute to the risk of breast cancer among Korean women. Promoting healthy behaviors throughout life may support long-term risk reduction.

## 1. Introduction

Breast cancer is the most frequently diagnosed cancer and is the leading cause of cancer-related mortality among women worldwide [1]. In South Korea, the incidence of breast cancer has steadily increased over the past two decades, becoming the most prevalent malignancy among women since 2019 [2]. According to recent national statistics, the age-standardized incidence of breast cancer in Korea reached 132.8 per 100,000 in 2021, a figure comparable to that in the United States (131.8 per 100,000) [2,3]. The increasing incidence of breast cancer has been attributed to several factors, including longer life expectancies, declining fertility rates, and the widespread adoption of Western lifestyles [4,5]. In Korea, these social and behavioral shifts have occurred rapidly owing to accelerated economic development [6]. Similar patterns have been observed in Asian migrant populations living abroad; for example, breast cancer risk among Asian–American women increases with greater acculturation and longer duration of residence in Western countries [7].

Several modifiable lifestyle and metabolic factors, including obesity, dyslipidemia, alcohol consumption, cigarette smoking, and the presence of diabetes mellitus (DM), have been implicated in the development of breast cancer [8,9]. Notably, many of these factors are established contributors to cardiovascular disease, a major cause of comorbidities and the second leading cause of death in patients with breast cancer [10]. Understanding the roles of these shared risk factors is critical for developing preventive strategies to address cancer and improve overall health. Although these associations have been studied in Western populations, evidence in Asian populations, particularly in Korea, remains limited. Given the unique epidemiological and sociodemographic characteristics of the Korean population, including an earlier peak age of breast cancer onset and historically lower obesity prevalence, further investigation is warranted.

Therefore, this study aimed to assess the association between health-related risk factors, including dyslipidemia, body weight, alcohol consumption, cigarette smoking, and DM, and the incidence of breast cancer in a large, nationally representative Korean cohort, using data from the National Health Insurance Service. Additionally, through age-stratified analyses, we examined potential differences in risk patterns across different life stages.

## 2. Materials and Methods

### 2.1. Study Population and Participant Selection

We used data from the Korean National Health Insurance Service (NHIS) database, which includes comprehensive information on sociodemographic factors, medical diagnoses, prescriptions, procedures, and health-screening data for the entire South Korean population, as described previously [11].

**Figure 1:**
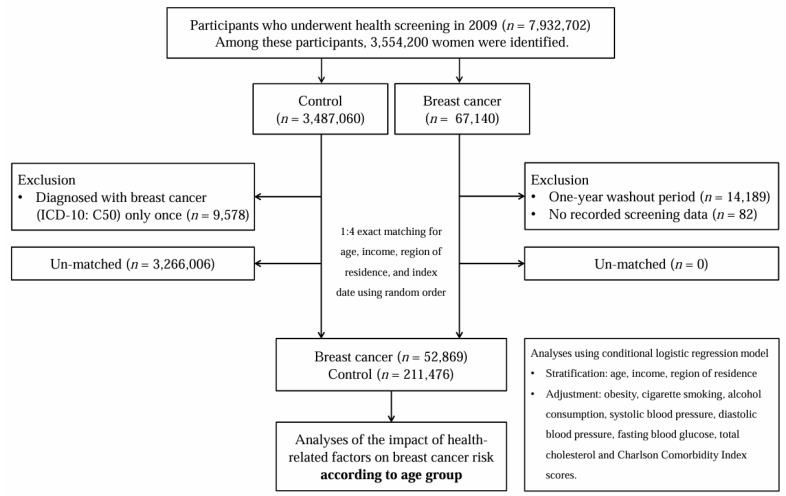
A schematic illustration of the participant selection process used in the present study. Of the 7,932,702 participants, 52,869 women with breast cancer and 211,476 control participants were included.

The NHIS provides a customized dataset that includes data on all adults who participated in the 2009 National Health Screening Program (*n* = 7,932,702). Of these, 3,554,200 women were included in the analysis. Participants diagnosed with breast cancer between 2010 and 2021 were identified based on the International Classification of Disease, tenth revision (ICD-10) diagnostic codes (C50) and special case registration codes (V193 or V194). To ensure the inclusion of only incident cases, participants diagnosed with breast cancer in 2009 were excluded as part of the one-year washout period. In addition, participants without complete screening data and those with a single claim for breast cancer were excluded. Women with no recorded diagnosis of breast cancer between 2009 and 2021 were included in the control group. Breast cancer cases were matched with controls in a 1:4 ratio based on age, income, and region of residence, using random sampling. Control participants were randomly selected to minimize selection bias. The index date for breast cancer cases was defined as the date of the first recorded treatment, and this date was applied to the matched controls. After matching, 52,869 breast cancer cases and 211,476 controls were included in the final analysis. Subsequently, data on health-related factors, including dyslipidemia, obesity, frequency of alcohol consumption, cigarette smoking, and the presence of DM, were collected (Figure 1).

### 2.2. Exposure Variables

Health-related exposures, including dyslipidemia, DM, body mass index (BMI), cigarette smoking, and alcohol consumption, were assessed before the index date. Dyslipidemia was defined as total cholesterol ≥ 200 mg/dL or ≥2 claims under ICD-10 code E78. DM was defined as fasting blood sugar ≥ 126 mg/dL or ≥2 claims under ICD-10 codes E11–E14. BMI was calculated from the two latest health screening data prior to the index date and categorized as underweight (<18.5 kg/m^2^), normal weight (18.5–22.9 kg/m^2^), overweight (23–24.9 kg/m^2^), obesity class I (25–29.9 kg/m^2^), and obesity class II (≥30 kg/m^2^), according to Korean guidelines [12,13]. Cigarette smoking status was categorized as nonsmoker, past smoker, or current smoker [13]. Alcohol consumption was categorized as <1 or ≥1 time/week.

### 2.3. Outcome Variable (Breast Cancer)

The primary outcome was breast cancer, defined by ≥ 2 claims under ICD-10 code C50 and the presence of a special case registration code (V193 or V194), to improve diagnostic accuracy.

### 2.4. Covariates

Sociodemographic covariates included age (categorized into 5-year intervals), income level (five classes), and region of residence (urban or rural) [14]. The clinical covariates included systolic and diastolic blood pressure, fasting blood sugar levels, and total cholesterol levels.

Comorbidity burden was evaluated using the Charlson Comorbidity Index (CCI), excluding cancer-related conditions to prevent outcome overlap. The CCI is a widely used tool for measuring overall disease burden using 17 comorbidities. A score was assigned to each participant depending on the disease severity and number. The CCI was measured as a continuous variable (0 [no comorbidities] to 29 [multiple comorbidities]) [15]. To avoid multicollinearity, total cholesterol was excluded from the models in which dyslipidemia was the primary exposure (Table 2), and fasting blood sugar was excluded when DM was the exposure of interest (Table 6).

### 2.5. Statistical Analysis

Descriptive characteristics were compared between groups using standardized differences. Associations between exposure and breast cancer were analyzed using conditional logistic regression to estimate odds ratios (ORs) and 95% confidence intervals (CIs). Both the unadjusted and adjusted models are presented. Subgroup analyses were conducted based on age group (<50 and ≥50 years of age). Statistical significance was defined as a two-sided *p* value of < 0.05. All the analyses were performed using SAS version 9.4 (SAS Institute Inc., Cary, NC, USA).

## 3. Results

### 3.1. Baseline Characteristics of the Participants

In total, 52,869 patients with breast cancer and 211,476 matched controls were included in the analyses. Matching at a 1:4 ratio based on age, income, and region of residence yielded well-balanced groups, as indicated by minimal standardized differences across covariates. The peak age group in both cohorts was 50–54 years (19.1%). The distribution of participants across age, income level, and region of residence was identical between groups, with standardized differences of 0.00 for each (Table 1).

### 3.2. Dyslipidemia and Breast Cancer Risk

Dyslipidemia was significantly associated with an increased risk of breast cancer. The adjusted OR (aOR) for breast cancer among individuals with dyslipidemia was 1.12 (95% CI, 1.10–1.14; *p* < 0.001), indicating a modest but statistically robust association. In subgroup analyses stratified by age, this association remained significant both among women younger than 50 years (aOR 1.16; 95% CI, 1.11–1.21) and those older than 50 years of age (aOR 1.10; 95% CI, 1.07–1.13) (Table 2).

### 3.3. BMI and Breast Cancer Risk

Compared to women with normal BMI (18.5–22.9 kg/m^2^), underweight participants (BMI < 18.5 kg/m^2^) had a significantly lower risk of breast cancer (aOR 0.93; 95% CI, 0.88–0.98; *p* = 0.015). In contrast, overweight (BMI 23–24.9 kg/m^2^), obesity class I (BMI 25–29.9 kg/m^2^), and obesity class II (BMI ≥ 30kg/m^2^) were all associated with an elevated risk of breast cancer (aORs: 1.02, 1.08, and 1.18, respectively; all *p* < 0.001).

Stratified analysis revealed a significant association with age. Among participants aged ≥ 50 years, a clear dose–response pattern was observed: overweight: aOR 1.04 (95% CI, 1.01–1.08; *p* = 0.005), obesity class I: aOR 1.14 (95% CI, 1.11–1.17; *p* < 0.001), and obesity class II: aOR 1.33 (95% CI, 1.26–1.41; *p* < 0.001).

In contrast, in women < 50 years of age, a higher BMI appeared to be inversely related to breast cancer: obesity class I: aOR 0.90 (95% CI, 0.86–0.95; *p* < 0.001) and obesity class II: aOR 0.78 (95% CI, 0.71–0.86; *p* < 0.001) (Table 3).

### 3.4. Alcohol Consumption and Breast Cancer Risk

No statistically significant association was found between frequency of alcohol consumption (≥ 1 or < 1 time/week) and breast cancer risk in the total study cohort (aOR 1.00; 95% CI, 0.98–1.02; *p* = 0.985), nor in any age subgroup (Table 4).

### 3.5. Cigarette Smoking and Breast Cancer Risk

No significant overall association was observed between cigarette smoking and incidence of breast cancer. However, in the subgroup of women aged ≥ 50 years, past or current smokers exhibited a slightly elevated risk compared to never-smokers (aOR 1.07; 95% CI, 1.00–1.14; *p* = 0.036) (Table 5).

### 3.6. Diabetes Mellitus and Breast Cancer Risk

In the total population, DM was not significantly associated with breast cancer risk (aOR 0.98; 95% CI, 0.95–1.01; *p* = 0.117). However, in women < 50 years of age, DM was inversely associated with breast cancer (aOR 0.87; 95% CI, 0.81–0.95; *p* < 0.001), while no significant association was found in women ≥ 50 years of age (Table 6).

### 3.7. Sensitivity Analysis and Model Diagnostics

To verify the robustness of the study findings, a sensitivity analysis was performed using an unconditional logistic regression model (Supplementary Table S1). The results were consistent with those obtained from the conditional logistic regression analysis, confirming the stability of the associations.

Multicollinearity among variables was assessed by calculating the variance inflation factors (VIFs) for all covariates (Supplementary Table S2). All VIF values were below the commonly accepted threshold, indicating no significant multicollinearity.

## 4. Discussion

In this large nationwide population-based cohort study, we investigated the association between health-related risk factors and incidence of breast cancer in Korean women. Dyslipidemia and obesity were significantly associated with an increased risk of breast cancer. Other risk factors, such as alcohol consumption, cigarette smoking, and DM, showed weaker or age-dependent associations. These findings provide important epidemiological insights into the evolving understanding of modifiable risk factors for breast cancer in East Asian populations.

We found that dyslipidemia was consistently associated with a modest increase in breast cancer risk across all age groups (95% CI, 1.10–1.14; *p* < 0.001). While the effect size was small (aOR = 1.12; 95% CI, 1.10–1.14; *p* < 0.001), this finding is noteworthy given the biologically plausible association between cholesterol metabolism and breast cancer development [16]. As a precursor of estrogen synthesis, cholesterol may promote the proliferation of hormone receptor-positive tumors; however, previous studies have reported inconsistent findings regarding this association [17]. While our findings support a potential link between dyslipidemia and breast cancer, further investigation is needed to clarify the mechanisms involved and assess whether lipid-lowering interventions could affect breast cancer risk.

The relationship between BMI and breast cancer demonstrated an age-dependent pattern. In women aged ≥ 50 years, a clear positive association was observed between an increased BMI and breast cancer risk. The aORs were 1.04 (95% CI, 1.01–1.08) for overweight (BMI ≥ 23 to < 25 kg/m^2^), 1.14 (95% CI, 1.11–1.17) for obesity class I (BMI ≥ 25 to < 30 kg/m^2^), and 1.33 (95% CI, 1.26–1.41) for obesity class II (BMI ≥ 30kg/m^2^), indicating a dose–response relationship between BMI and breast cancer risk. This finding is consistent with existing literature, as demonstrated by a meta-analysis of 50 studies that reported that overweight and obesity increased breast cancer risk by 1.12-fold and 1.16-fold, respectively [18]; however, most of these studies did not distinguish between higher BMI categories, often combining obesity class I (BMI ≥ 25 to < 30 kg/m^2^) and obesity class II (BMI ≥ 30 kg/m^2^). Increased aromatase activity in the adipose tissue activity after menopause can lead to elevated circulating estrogen levels, which may promote the development of hormone-sensitive breast tumors [19,20]. Obesity may also influence breast cancer development through various other mechanisms, including activation of the PI3K/Akt/mTOR signaling pathway, upregulation of leptin, downregulation of adiponectin, increased insulin-like growth factor signaling, and elevated proinflammatory cytokine production [19,21].

In contrast, among women younger than 50 years, a higher BMI was inversely associated with breast cancer risk in our study. In this subgroup, the aORs for obesity class I (BMI ≥ 25 to < 30 kg/m^2^) and obesity class II (BMI ≥ 30 kg/m^2^) were 0.90 (95% CI, 0.86–0.95) and 0.78 (95% CI, 0.71–0.86), respectively. This paradoxical relationship has been observed in previous studies [18] and may be partially attributed to hormonal differences. In premenopausal women, obesity may lead to more frequent anovulatory cycles, resulting in lower cumulative exposure to estrogen and progesterone [22]. However, other possible explanations, including detection bias attributed to lower screening rates in obese women or differences in tumor biology, have also been suggested [23]. Although the protective association observed in younger women warrants further investigation, maintaining a healthy weight throughout life is advisable, especially considering the risk of weight gain during menopause and its potential long-term implications [24].

Our study found no consistent association between alcohol consumption, cigarette smoking, the presence of DM and breast cancer in the overall population. These findings differ slightly from those of previous studies. Although previous meta-analyses have reported a dose-dependent relationship between alcohol consumption and breast cancer [25], the binary categorization of alcohol intake in our dataset may have obscured subtle associations. Similarly, cigarette smoking had no significant effects overall. Although the association between cigarette smoking and breast cancer is still debated [26], a recent meta-analysis demonstrated a modest increase in the risk of breast cancer among smokers [27]. Notably, in our subgroup analysis, cigarette smoking was associated with an elevated breast cancer risk in women ≥ 50 years of age (aOR, 1.07; 95% CI, 1.00–1.14), which could be attributed to cumulative lifetime exposure to tobacco-related carcinogens [27,28]. The absence of a significant association in the full cohort may result from issues such as under-reported lifestyle behaviors or limited granularity in the exposure data.

Although previous studies have linked DM to breast cancer incidence, particularly among postmenopausal women [29,30], our findings demonstrated inconsistent associations. One possible explanation for this is that the effect of DM may have been masked by the stronger effects of BMI and other covariates in our model. Alternatively, the definition of DM based on claims data may not fully capture glycemic control or disease duration.

Taken together, our findings provide additional context for understanding breast cancer risk factors in the Korean population, particularly considering the shifting demographic and metabolic profiles of the country. Breast cancer incidence peaks in Korean women at a younger age (50–54 years), earlier than in any other Asian or Western country [31]. While obesity rates in Korea have historically been lower (151 out of 200 countries) than those in Western populations [32], they have steadily increased over the past decade [13]. Therefore, continued monitoring of metabolic health and its relationship with cancer risk is warranted.

This study has some limitations. First, the study population consisted of individuals who participated in a national health-screening program, which may have introduced a selection bias. Additionally, because lifestyle-related risk factors were self-reported, some degree of information bias might have been present. Second, the analysis was based on baseline measurements; therefore, we could not account for changes in BMI, lipid profiles, or lifestyle behaviors over time. Third, our dataset lacked detailed clinical information on breast cancer subtypes such as hormone receptor status and molecular classification, which limited our ability to assess subtype-specific risk patterns. Finally, certain confounders, such as reproductive history, family history of breast cancer, and use of hormone replacement therapy, were unavailable and may have influenced the observed associations.

Nevertheless, this study has several methodological strengths that enhanced the reliability and relevance of its findings. First, we used a large nationally representative cohort from the Korean National Health Insurance Service database that included detailed clinical and demographic information. The use of this comprehensive database enabled a robust analysis with excellent generalizability to a broader Korean female population. Second, the long follow-up period (up to 12 years) facilitated the identification of incident breast cancer cases while establishing a clear temporal relationship between exposure and outcome. By applying a washout period in 2009, we strengthened the case identification by ensuring that only newly diagnosed cases were included, thereby minimizing the risk of reverse causation. Third, a 1:4 matching was performed between patients with breast cancer and controls based on key demographic variables (age, income, and region), reducing potential selection bias and improving internal validity. We also employed multiple conditional logistic regression models to adjust for a wide range of clinical and lifestyle covariates and to enhance the robustness of the associations. Fourth, this study examined multiple modifiable health-related factors, including BMI, dyslipidemia, cigarette smoking, alcohol consumption, and the presence of DM, within a single analytical framework. This allowed us to analyze how these factors, independently or in combination, contribute to the risk of developing breast cancer. Finally, our analysis included age-stratified subgroup evaluations that revealed distinct patterns of association across different life stages. These findings provide a more nuanced understanding of how metabolic and lifestyle factors influence breast cancer risk depending on age and could support the development of personalized preventive approaches.

## 5. Conclusions

Obesity and dyslipidemia are significant risk factors for breast cancer in Korean women. Postmenopausal women demonstrated a clear positive association with increased BMI, whereas premenopausal women showed an inverse association. These findings highlight the importance of tailoring preventive strategies to consider metabolic health and age. Further longitudinal mechanistic studies are warranted to enhance our understanding and inform evidence-based cancer prevention policies in Asian populations.

## Figures and Tables

**Table 1:** General characteristics of the participants.

Characteristics		Total Participants		
		Breast Cancer	Control	Standardized Difference
Total number (*n*, %)		52,869 (100.0)	211,476 (100.0)	
Age, years (*n*, %)				0.00
	20–24	4 (0.01)	16 (0.01)	
	25–29	145 (0.27)	580 (0.27)	
	30–34	832 (1.57)	3328 (1.57)	
	35–39	2243 (4.24)	8972 (4.24)	
	40–44	4672 (8.84)	18,688 (8.84)	
	45–49	8625 (16.31)	34,500 (16.31)	
	50–54	10,108 (19.12)	40,432 (19.12)	
	55–59	8309 (15.72)	33,236 (15.72)	
	60–64	7259 (13.73)	29,036 (13.73)	
	65–69	4507 (8.52)	18,028 (8.52)	
	70–74	3367 (6.37)	13,468 (6.37)	
	75–79	1742 (3.29)	6968 (3.29)	
	≥ 80	1056 (2.00)	4224 (2.00)	
Income (*n*, %)				0.00
	1 (lowest)	13,918 (26.33)	55,672 (26.33)	
	2	11,011 (20.83)	44,044 (20.83)	
	3	7548 (14.28)	30,192 (14.28)	
	4	9225 (17.45)	36,900 (17.45)	
	5 (highest)	11,167 (21.12)	44,668 (21.12)	
Region of residence (*n*, %)				0.00
	Urban	25,262 (47.78)	101,048 (47.78)	
	Rural	27,607 (52.22)	110,428 (52.22)	
Obesity * (*n*, %)				0.07
	Underweight	1576 (2.98)	7005 (3.31)	
	Normal	21,663 (40.97)	90,022 (42.57)	
	Overweight	12,335 (23.33)	50,256 (23.76)	
	Obesity class I	14,634 (27.68)	55,251 (26.13)	
	Obesity class II	2661 (5.03)	8942 (4.23)	
Smoking status (*n*, %)				0.00
	Never	50,574 (95.66)	202,582 (95.79)	
	Former	911 (1.72)	3389 (1.60)	
	Current	1384 (2.62)	5505 (2.60)	
Alcohol consumption (*n*, %)				0.00
	< 1 time a week	40,800 (77.17)	162,937 (77.05)	
	≥ 1 time a week	12,069 (22.83)	48,539 (22.95)	
SBP, Mean ± SD		121.49 ± 15.30	120.93 ± 15.25	0.04
DBP, Mean ± SD		74.74 ± 9.85	74.62 ± 9.87	0.01
FBS, Mean ± SD		98.80 ± 22.00	97.88 ± 21.66	0.02
Total cholesterol, Mean ± SD		198.55 ± 38.69	199.57 ± 38.54	0.02
CCI score, Mean ± SD		0.38 ± 0.76	0.27 ± 0.71	0.14
Dyslipidemia (*n*, %)		24,996 (47.28)	94,048 (44.47)	0.06
Diabetes mellitus (*n*, %)		9366 (17.72)	34,543 (16.33)	0.04

Abbreviations: CCI, Charlson Comorbidity Index; SBP, Systolic blood pressure; DBP, Diastolic blood pressure; FBS, Fasting blood sugar; SD, Standard deviation; * Obesity (BMI, body mass index, kg/m^2^) was categorized as <18.5 (underweight), ≥18.5 to <23 (normal), ≥23 to <25 (overweight), ≥25 to <30 (obesity class I), and ≥30 (obesity class II).

**Table 2:** Crude and adjusted odds ratios of dyslipidemia for breast cancer with subgroup analyses by age.

Characteristics		Breast Cancer	Control	Odds Ratios (95% Confidence Interval)			
		(Exposure/Total, %)	(Exposure/Total, %)	Crude ^†^	*p* Value	Adjusted ^†‡^	***p* Value**
Total participants (*n* = 264,345)							
	Non-dyslipidemia	27,873/52,869 (52.7)	117,428/211,476 (55.5)	1		1	
	Dyslipidemia	24,996/52,869 (47.3)	94,048/211,476 (44.5)	1.12 (1.10–1.14)	<0.001 *	1.12 (1.10–1.14)	<0.001 *
Participants < 50 years of age (*n* = 82,605)							
	Non-dyslipidemia	12,703/16,521 (76.9)	52,543/66,084 (79.5)	1		1	
	Dyslipidemia	3818/16,521 (23.1)	13,541/66,084 (20.5)	1.13 (1.11–1.16)	<0.001 *	1.16 (1.11–1.21)	<0.001 *
Participants ≥ 50 years of age (*n* = 181,740)							
	Non-dyslipidemia	15,170/36,348 (41.7)	64,885/145,392 (44.6)	1		1	
	Dyslipidemia	21,178/36,348 (58.3)	80,507/145,392 (55.4)	1.17 (1.12–1.22)	<0.001 *	1.10 (1.07–1.13)	<0.001 *

* Conditional logistic regression model, significance at *p* < 0.05; ^†^ Models were stratified by age, income, and region of residence. ^‡^ The model was adjusted for obesity, smoking, alcohol consumption, SBP, DBP, fasting blood sugar, and CCI scores.

**Table 3:** Crude and adjusted odds ratios of obesity for breast cancer with subgroup analyses by age.

Characteristics		Breast Cancer	Control	Odds Ratios (95% Confidence Interval)			
		(Exposure/Total, %)	(Exposure/Total, %)	Crude ^†^	*p* Value	Adjusted ^†‡^	*p* Value
Total participants (*n* = 264,345)							
	Underweight	1576/52,869 (3.0)	7005/211,476 (3.3)	0.93 (0.88–0.99)	0.020 *	0.93 (0.88–0.98)	0.015 *
	Normal	21,663/52,869 (41.0)	90,022/211,476 (42.6)	1		1	
	Overweight	12,335/52,869 (23.3)	50,256/211,476 (23.8)	1.02 (1.00–1.05)	0.116	1.02 (0.99–1.04)	0.512
	Obesity I	14,634/52,869 (27.7)	55,251/211,476 (26.1)	1.10 (1.08–1.13)	<0.001 *	1.08 (1.06–1.11)	<0.001 *
	Obesity II	2661/52,869 (5.0)	8942/211,476 (4.2)	1.24 (1.18–1.29)	<0.001 *	1.18 (1.12–1.23)	<0.001 *
Participants < 50 years of age (*n* = 82,605)							
	Underweight	998/16,521 (6.0)	3898/66,084 (5.9)	1.01 (0.94–1.09)	0.842	1.01 (0.94–1.09)	0.817
	Normal	9039/16,521 (54.7)	35,500/66,084 (53.7)	1		1	
	Overweight	3138/16,521 (19.0)	12,645/66,084 (19.1)	0.97 (0.93–1.02)	0.251	0.96 (0.91–1.00)	0.057
	Obesity class I	2812/16,521 (17.0)	11,609/66,084 (17.6)	0.95 (0.91–1.00)	0.035 *	0.90 (0.86–0.95)	<0.001 *
	Obesity class II	534/16,521 (3.2)	2432/66,084 (3.7)	0.86 (0.78–0.95)	0.003	0.78 (0.71–0.86)	<0.001 *
Participants ≥ 50 years of age (*n* = 181,740)							
	Underweight	578/36,348 (1.6)	3107/145,392 (2.1)	0.80 (0.73–0.88)	<0.001 *	0.81 (0.74–0.89)	<0.001 *
	Normal	12,624/36,348 (34.7)	54,522/145,392 (37.5)	1		1	
	Overweight	9197/36,348 (25.3)	37,611/145,392 (25.9)	1.06 (1.03–1.09)	<0.001 *	1.04 (1.01–1.08)	0.005 *
	Obesity class I	11,822/36,348 (32.5)	43,642/145,392 (30.0)	1.18 (1.14–1.21)	<0.001 *	1.14 (1.11–1.17)	<0.001 *
	Obesity class II	2127/36,348 (5.9)	6510/145,392 (4.5)	1.42 (1.35–1.50)	<0.001 *	1.33 (1.26–1.41)	<0.001 *

* Conditional logistic regression model, significance at *p* < 0.05; ^†^ Models were stratified by age, income, and region of residence. ^‡^ The model was adjusted for smoking, alcohol consumption, SBP, DBP, fasting blood sugar, total cholesterol, and CCI scores.

**Table 4:** Crude and adjusted odds ratios of alcohol consumption (< 1/week vs. ≥ 1/week) for breast cancer with subgroup analyses by age.

Characteristics		Breast Cancer	Control	Odds Ratios (95% Confidence Interval)			
		(Exposure/Total, %)	(Exposure/Total, %)	Crude ^†^	*p* Value	Adjusted ^†‡^	*p* Value
Total participants (*n* = 264,345)							
	<1/week	40,800/52,869 (77.2)	162,937/211,476 (77.0)	1		1	
	≥1/week	12,069/52,869 (22.8)	48,539/211,476 (23.0)	0.99 (0.97–1.02)	0.543	1.00 (0.98–1.02)	0.985
Participants < 50 years of age (*n* = 82,605)							
	<1/week	10,646/16,521 (64.4)	42,203/66,084 (63.9)	1		1	
	≥1/week	5875/16,521 (35.6)	23,881/66,084 (36.1)	0.98 (0.94–1.01)	0.165	0.99 (0.95–1.02)	0.414
Participants ≥ 50 years of age (*n* = 181,740)							
	<1/week	30,154/36,348 (83.0)	120,734/145,392 (83.0)	1		1	
	≥1/week	6194/36,348 (17.0)	24,658/145,392 (17.0)	1.01 (0.98–1.04)	0.707	1.01 (0.98–1.04)	0.509

^†^ Models were stratified by age, income, and region of residence. ^‡^ The model was adjusted for obesity, smoking, SBP, DBP, fasting blood sugar, total cholesterol, and CCI scores.

**Table 5:** Crude and adjusted odds ratios of cigarette smoking (never vs. former/current) for breast cancer with subgroup analyses by age.

Characteristics		Breast Cancer	Control	Odds Ratios (95% Confidence Interval)			
		(Exposure/Total, %)	(Exposure/Total, %)	Crude ^†^	*p* Value	Adjusted ^†‡^	*p* Value
Total participants (*n* = 264,345)							
	Never	50,574/52,869 (95.7)	202,582/211,476 (95.8)	1		1	
	Former/current	2295/52,869 (4.3)	8894/211,476 (4.2)	1.03 (0.99–1.08)	0.163	1.01 (0.97–1.06)	0.608
Participants < 50 years of age (*n* = 82,605)							
	Never	15,562/16,521 (94.2)	62,107/66,084 (94.0)	1		1	
	Former/current	959/16,521 (5.8)	3977/66,084 (6.0)	0.96 (0.90–1.04)	0.299	0.94 (0.88–1.02)	0.128
Participants ≥ 50 years of age (*n* = 181,740)							
	Never	35,012/36,348 (96.3)	140,475/145,392 (96.6)	1		1	
	Former/current	1336/36,348 (3.7)	4917/145,392 (3.4)	1.09 (1.03–1.16)	0.006 *	1.07 (1.00–1.14)	0.036 *

* Conditional logistic regression model, significance at *p* < 0.05; ^†^ Models were stratified by age, income, and region of residence. ^‡^ The model was adjusted for obesity, alcohol consumption, SBP, DBP, fasting blood sugar, total cholesterol, and CCI scores.

**Table 6:** Crude and adjusted odds ratios of DM for breast cancer with subgroup analyses by age.

Characteristics		Breast Cancer	Control	Odds Ratios (95% Confidence Interval)			
		(Exposure/Total, %)	(Exposure/Total, %)	Crude ^†^	*p* Value	Adjusted ^†‡^	*p* Value
Total participants (*n* = 264,345)							
	Non-DM	43,503/52,869 (82.3)	176,933/211,476 (83.7)	1		1	
	DM	9366/52,869 (17.7)	34,543/211,476 (16.3)	1.10 (1.08–1.13)	<0.001 *	0.98 (0.95–1.01)	0.117
Participants < 50 years of age (*n* = 82,605)							
	Non-DM	15,565/16,521 (94.2)	62,436/66,084 (94.5)	1		1	
	DM	956/16,521 (5.8)	3648/66,084 (5.5)	1.05 (0.98–1.13)	0.181	0.87 (0.81–0.95)	<0.001 *
Participants ≥ 50 years of age (*n* = 181,740)							
	Non-DM	27,938/36,348 (76.9)	114,497/145,392 (78.8)	1		1	
	DM	8410/36,348 (23.1)	30,895/145,392 (21.2)	1.12 (1.09–1.16)	<0.001 *	1.00 (0.97–1.03)	0.913

Abbreviations: DM, Diabetes mellitus; * Conditional logistic regression model, significance at *p* < 0.05; ^†^ Models were stratified by age, income, and region of residence. ^‡^ The model was adjusted for obesity, smoking, alcohol consumption, SBP, DBP, total cholesterol, and CCI scores.

## Data Availability

Data are available from the authors upon request.

## Supplementary Materials

The following supporting information can be downloaded at: https://www.mdpi.com/article/10.3390/jcm14217816/s1, Supplementary Table S1: Sensitivity analysis using an unconditional logistic regression model; Supplementary Table S2: Assessment of multicollinearity using variance inflation factor.

## Abbreviations

The following abbreviations are used in this manuscript:

aOR	Adjusted odds ratio
BMI	Body mass index
CCI	Charlson comorbidity index
DBP	Diastolic blood pressure
DM	Diabetes mellitus
FBS	Fasting blood sugar
SBP	Systolic blood pressure
SD	Standard deviation
VIF	Variance inflation factor
