# Supplementary material for: Postmenopausal Obesity and Dyslipidemia as Risk Factors for Breast Cancer in Korean Women: Analysis of a National Health Screening Cohort
Source: J Clin Med. 2025 Nov 3;14(21):7816. doi: 10.3390/jcm14217816 (PMC12608468; doi:10.3390/jcm14217816)
Supplement: Supplementary file 1 [file jcm-14-07816-s001.zip › Supplementary Table S1 (unconditional logistic regression).pdf]

Supplementary Table S1. Sensitivity analysis using an unconditional logistic regression model.

Characteristics	Breast cancer	Control	Odd ratios (95% confidence interval)			
	(exposure/total, %)	(exposure/total, %)	Crude	P-value	Adjusted †	*p*-value
Dyslipidemia						
Non-dyslipidemia	27,873/52,869 (52.7)	117,428/211,476 (55.5)	1		1	
Dyslipidemia	24,996/52,869 (47.3)	94,048/211,476 (44.5)	1.12 (1.10–1.14)	< 0.001*	1.16 (1.13–1.18)	< 0.001*
Obesity						
Underweight	1,576/52,869 (3.0)	7,005/211,476 (3.3)	0.94 (0.88–0.99)	0.019*	0.92 (0.87–0.98)	0.005*
Normal	21,663/52,869 (41.0)	90,022/211,476 (42.6)	1		1	
Overweight	12,335/52,869 (23.3)	50,256/211,476 (23.8)	1.02 (1.00–1.05)	0.116	1.01 (0.99–1.04)	0.279
Obesity class I	14,634/52,869 (27.7)	55,251/211,476 (26.1)	1.10 (1.08–1.13)	< 0.001*	1.07 (1.05–1.10)	< 0.001*
Obesity class II	2,661/52,869 (5.0)	8,942/211,476 (4.2)	1.24 (1.18–1.30)	< 0.001*	1.16 (1.11–1.22)	< 0.001*
Alcohol consumption						
< 1/week	40,800/52,869 (77.2)	162,937/211,476 (77.0)	1		1	
≥ 1/week	12,069/52,869 (22.8)	48,539/211,476 (23.0)	0.99 (0.97–1.02)	0.543	1.00 (0.98–1.02)	0.908
Smoking status						
Never	50,574/52,869 (95.7)	202,582/211,476 (95.8)	1		1	
Former/Current	2,295/52,869 (4.3)	8,894/211,476 (4.2)	1.03 (0.99–1.08)	0.163	1.01 (0.97-1.06)	0.628
Diabetes mellitus						
Non-DM	43,503/52,869 (82.3)	176,933/211,476 (83.7)	1		1	
DM	9,366/52,869 (17.7)	34,543/211,476 (16.3)	1.10 (1.08–1.13)	< 0.001*	0.95 (0.92–0.98)	0.001*

Abbreviations: SBP, Systolic blood pressure; DBP, Diastolic blood pressure; FBG. Fasting blood glucose; CCI, Charlson Comorbidity Index; DM, Diabetes mellitus

* Unconditional logistic regression model, Significance at $p < 0.05$

† The model was adjusted for age, income, and region of residence, smoking, alcohol consumption, obesity, SBP, DBP, fasting blood glucose, total cholesterol, and CCI scores.
