# Supplementary material for: Postmenopausal Obesity and Dyslipidemia as Risk Factors for Breast Cancer in Korean Women: Analysis of a National Health Screening Cohort
Source: J Clin Med. 2025 Nov 3;14(21):7816. doi: 10.3390/jcm14217816 (PMC12608468; doi:10.3390/jcm14217816)
Supplement: Supplementary file 1 [file jcm-14-07816-s001.zip › Supplementary Table S2 (Multicollinearity).pdf]

Supplementary Table S2. Assessment of multicollinearity using variance inflation factor (VIF)

Variable	Total
Dyslipidemia	1.10948
Obesity	1.07447
Alcohol consumption	1.04933
Smoking status	1.02471
Diabetes mellitus	1.46809

Note: All other covariates had VIF values between 1.001 and 2.17, indicating no multicollinearity concern.
